# Growth hormone therapy response in children with short stature

**DOI:** 10.1186/s43054-023-00173-y

**Published:** 2023-06-05

**Authors:** Amira Ahmed Gad, Radwa Shamma, Mohamed A. Elmonem, Nora E. Badawi, Lubna Fawaz, Mona Mamdouh Hassan

**Affiliations:** 1 Diabetes, Endocrine, and Metabolism Pediatric Unit (DEMPU), Pediatric Department, Cairo University Children Hospital, 1 Ali Ibrahim Pasha Street, Cairo, 11617 Egypt; 2 Department of Clinical and Chemical Pathology, Faculty of Medicine, Cairo University, Cairo, Egypt; 3 Egypt Center of Research and Regenerative Medicine (ECRRM), Cairo, Egypt; 4 School of Medicine, Newgiza University, Cairo, Egypt

**Keywords:** Short stature, Growth hormone deficiency, Idiopathic short stature, Small for gestational age, Recombinant growth hormone, Height gain

## Abstract

**Background:**

Short stature is one of the main causes of children referral to pediatric endocrinologists. Common etiologies include idiopathic growth hormone deficiency (IGHD), small for gestational age (SGA), and idiopathic short stature (ISS).

**Objectives:**

The aim of this study was to assess and compare the response of children with IGHD, ISS, and SGA to growth hormone (GH) therapy.

**Methods:**

This was a mixed cohort study that included 40 children with short stature (classified into IGHD, ISS, and SGA) following up at Diabetes, Endocrine, and Metabolism Pediatric Unit (DEMPU), Cairo University Children’s Hospital. Ages ranged between 3 and 18 years. Recruited cases were evaluated for their 1-year response to GH therapy. In addition to history taking, physical examination, and anthropometric measurements, serum levels of IGF-1 were assayed at recruitment.

**Results:**

Among the 3 groups, height gain (cm/year) was significantly higher in the IGHD group (6.59 cm/year), followed by the ISS (4.63 cm/year) and SGA groups (4.46 cm/year) (*p* = 0.039). Using the Bang criterion for first-year responsiveness to GH therapy, most cases (30/40, 75%) were considered poor responders.

**Conclusion:**

There is a male predominance in children seeking medical advice for short stature. Starting GH therapy at an older age was associated with poor response. Children with IGHD respond better to GH therapy than those with ISS and SGA.

## Background

Short stature is an essential cause of referral to pediatric endocrinologists. It is defined as a height that is more than two standard deviations below the reference population’s mean height for age and sex. It is estimated to affect approximately 3% of children in a population [1]. It is considered a disabling condition that represents a psychological burden not only for the child but for his parents as well.

Short stature is a global health problem. In the USA, 2.2 million children less than 18 years of age have heights below the third percentile [2]. In Egypt, one of the developing countries, short stature is a major health burden especially among children less than 5 years [3].

Short stature can be due to various etiologies either primary or secondary growth disorder or idiopathic [4]. Growth hormone deficiency (GHD) is described as insufficient production of GH by the pituitary gland [5]. Idiopathic short stature (ISS) cases have normal birth weight and normal levels of GH secretion and no recognizable abnormalities to explain the child’s growth pattern [6]. A child born small for gestational age (SGA) is defined as having a birth weight or birth length less than two standard deviations for gestational age [7].

Only patients with established and approved indications for GH therapy should be offered it. Growth hormone deficiency, Turner syndrome, Prader-Willi syndrome, being small for gestational age, chronic renal insufficiency, and idiopathic short stature are all common pediatric indications for GH use [8].

## Objectives

The purpose of this study was to evaluate and compare the first-year response to growth hormone therapy and identify patients with poor response among those with idiopathic growth hormone deficiency, idiopathic short stature, and small for gestational age. This can offer a clue to revise the diagnosis, adjust growth hormone doses, or stop injections altogether in order to avoid unnecessary costs and injections.

## Methods

In this mixed cohort study, the anthropometric data and first year response to GH of 54 recruited cases were studied.

Included cases were cases with short stature (height below two SD for age and gender compared to the reference population), from both sexes, with age ranging from 3 to 18 years and receiving GH for a period of 1 year or less.

Patients with chromosomal anomalies, chronic medical disorders affecting linear growth, and those diagnosed with panhypopituitarism either congenital or acquired were excluded. Cases receiving GH for more than 1 year and GH-naive cases were also excluded. The study was conducted over a period of 1 year, from October 2020 to October 2021.

Data collection was done prospectively for cases not yet initiated on GH treatment and for cases receiving GH for less than 1 year duration (till completing first year treatment). Data collection from patients’ records was done retrospectively for cases that have been receiving GH treatment for 1 year duration.

The recruited cases were divided into 3 groups (IGHD, ISS, and SGA) based on the etiology of short stature and results of GH provocation tests. IGHD cases had peak GH value below 7 ng/dl in 2 growth hormone provocation tests. ISS cases had peak GH above 7 ng/ml in at least one of two GH provocation tests after exclusion of other causes of short stature. SGA cases had birth weight less than 2 standard deviation for gestational age.

All patients were subjected to full history taking and examination including anthropometric measurements. The Anthro-Calc application was used in calculating height and weight *Z* score. Height and weight SD for children ages 0–19 years in addition to adult height mean and SD values were calculated using WHO growth charts for Canada [9]. Mid-parental height and mid-parental height *Z* score calculation *r* (P, P) and *r* (P.O) were assumed to be 0.27 and 0.57 respectively [10].

Bang and Ranke criteria were used to classify cases in their first year of GH therapy into good and poor responders. Bang criterion (applied on all cases receiving GH for 1 year) defines poor responders as having change in height SDS in the 1st year less than 0.5 SDS [11]. “Ranke criterion” (applied only on patients with peak growth hormone less than 7 ng/ml in 2 provocation tests) defines poor response as change in height SDS in the 1st year less than 0.4 SDS if severe GHD (peak GH in 2 GH provocation tests < 5 ng/ml) but less than 0.3 SDS if less severe GHD (peak GH in 2 GH provocation tests 5–7 ng/ml) [12].

Previous investigations were obtained from medical records including thyroid profile and initial bone age before the start of GH treatment, which was done by plain x-ray of the left hand and wrist with interpretation of age using Greulich and Pyle atlas for bone age [13].

Growth hormone was analyzed by the automated electrochemiluminescence analyzer: Cobas e411, Roche Diagnostics, Germany. Interpretation of growth hormone provocation tests was done using new cut-off limit of 7.09 μg/l using the iSYS assay: GH peak value 7.09 ng/mL (7.09 μg/L) or more was considered normal, while GH peak value less than 7.09 ng/mL was considered subnormal [14]. Measurement of IGF-1 by ELISA technique was done for all cases at time of recruitment. Classification of IGF-1 level into normal and abnormal was done by comparing results of IGF-1 done in the present study to reference values done for age and sex by Ertl et al. [15].

The study was approved by the Ethical committee of the Faculty of Medicine, Cairo University (Approval code#MD-161–2021). Informed consent was obtained from all included patients by one of the legal guardians in addition to assent from children 8 years or above.

Data were statistically described in terms of mean ± standard deviation (± SD), median and interquartile range, or frequencies (number of cases) and percentages when appropriate. Numerical data were tested for the normal assumption using Kolmogorov–Smirnov test. Comparison of numerical variables between the study groups was done using Mann–Whitney *U* test for independent samples. For comparing categorical data, chi-square (*χ*^2^) test was performed. Correlation between various variables was done using Spearman rank correlation coefficient. Two-sided* p* values less than 0.05 were considered statistically significant. IBM SPSS (Statistical Package for the Social Science; IBM Corp, Armonk, NY, USA) release 22 for Microsoft Windows was used for all statistical analyses.

## Results

**Fig. 1:**
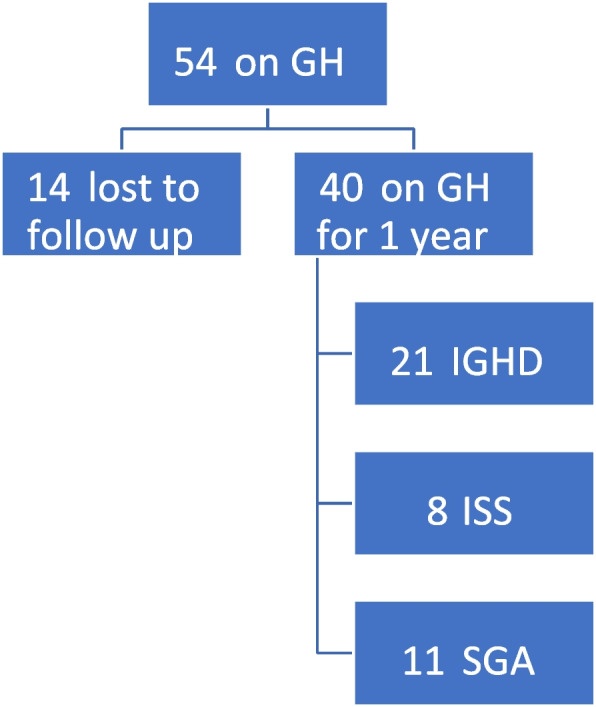
Classification of 54 recruited cases

Fifty-four cases, which were under national health insurance coverage and receiving GH therapy, were recruited. Only 40 cases (74%) continued follow-up in our study for 1 year, whereas the remaining 14 cases (26%) were lost to follow-up. The 40 cases were classified into 21 cases (52.5%) IGHD, 8 cases (20%) ISS, and 11 cases (27.5%) SGA (refer to Fig. 1).

**Fig. 2:**
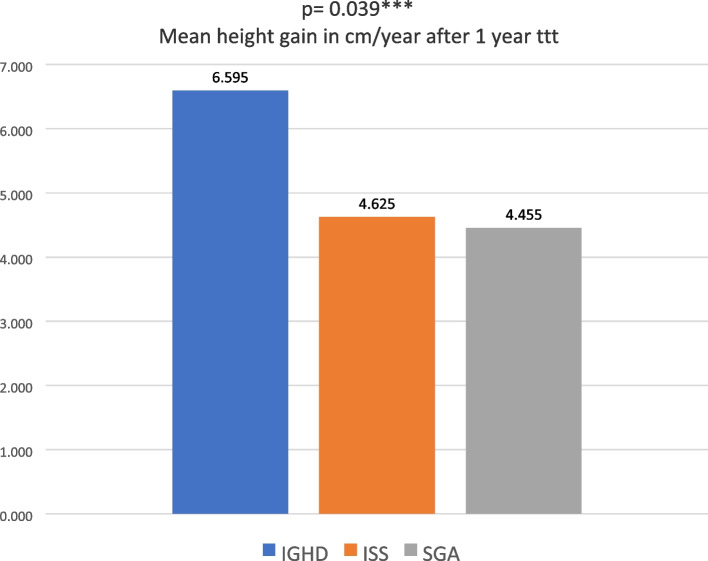
Comparing mean height gain (cm/year) after 1 year GH treatment among 3 groups

Tables 1 and 2 illustrate the main anthropometric data of the 40 cases on GH therapy before and after 1 year treatment respectively. The highest mean height gain (cm/year) was observed in IGHD group (6.595 cm/year), followed by ISS group (4.63 cm/year), and then SGA group (4.46 cm/year). This was statistically significant with (*p* = 0.039), as illustrated in Fig. 2.

**Table 1:** Comparison of age, anthropometric parameters and peak GH levels after provocation in the 3 studied groups (IGHD, ISS and SGA) (no. = 40)

Characteristics	IGHD (*n* = 21)	ISS (*n* = 8)	SGA (*n* = 11)	*p* value
Initial age (years)	12.56 ± 2.71	13.75 ± 3.52	11.34 ± 2.99	0.163
Initial height (cm)	129.1 ± 13.62	133.95 ± 17.21	123.27 ± 15.7	0.322
Initial height SD	-3.46 ± 0.58	-3.39 ± 0.56	-3.42 ± 0.78	0.931
Initial weight (kg)	29.57 ± 9.51	32.69 ± 12.92	26.73 ± 9.54	0.531
Initial weight SD	-2.56 ± 1.12	-2.875 ± 1.03	-2.51 ± 1.061	0.704
Maternal height (cm)	153.69 ± 3.65	155.25 ± 6.09	155.59 ± 5.63	0.729
Paternal height (cm)	166.88 ± 6.39	163.5 ± 6.46	167.77 ± 6.95	0.38
MPH (cm)	164.51 ± 7.3	164.06 ± 8.31	164.7 ± 9.3	0.901
MPH SD	-0.99 ± 0.398	-1.07 ± 0.52	-0.85 ± 0.55	0.359
Initial bone age (years)	8.84 ± 3.12	10.68 ± 2.095	8.3 ± 3.55	0.282
Peak GH in Clonidine test(ng/ml)	3.18 ± 1.53	12.66 ± 4.14	6.34 ± 6.02	ND
Peak GH in ITT (ng/ml)	3.28 ± 1.89	10.8 ± 6.24	4.89 ± 6.82	ND

*Cm* Centimetre, *SD* Standard deviation, *ng* Nanograms, *ml* Millilitre, *MPH* Mid-parental height, *GH* Growth hormone, *ITT* Insulin tolerance test, *IGHD* Idiopathic growth hormone deficiency, *ISS* Idiopathic short stature, *SGA* Small for gestational age, *ND* Not determined

**Table 2:** Comparison of anthropometric parameters, mean GH dose and mean height gain after 1 year GH therapy (no. = 40)

Characteristics (after 1^st^ year of GH therapy)	IGHD (*n* = 21) (Mean ± SD)	ISS (*n* = 8) (Mean ± SD)	SGA (*n* = 11) (Mean ± SD)	*p* value
Age (years)	13.56 ± 2.71	14.75 ± 3.52	12.34 ± 2.99	0.16
Height(cm)	135.26 ± 13.67	138.7 ± 16.78	128 ± 15.84	0.308
Height SD	-3.12 ± 0.75	-3.095 ± 0.78	-3.27 ± 0.92	0.976
Mean GH dose during the 1^st^ year (mg/kg/day)	0.04 ± 0.01	0.042 ± 0.02	0.039 ± 0.01	0.712
Mean height gain during the 1^st^ year (cm/year)	6.595 ± 2.39	4.63 ± 2.36	4.46 ± 2.14	0.039**
Δ height SD after 1^st^ year of GH therapy	0.34 ± 0.42	0.3 ± 0.45	0.15 ± 0.37	0.42

*1*^*st*^ First, *SD* Standard deviation, *cm* Centimetre, *MPH* Mid-parental height, *GH* Growth hormone, *mg* Milligram, *cm* Centimetre, Δ *height SD* Height SD after 1 year-initial height SD, *IGHD* Idiopathic growth hormone deficiency, *ISS* Idiopathic short stature, *SGA* Small for gestational age

^**^significant *p* value, 0.05

The mean GH dose during the first year of GH treatment was highest among cases with ISS (41.6 μg/kg/day), followed by IGHD (40.79 μg/kg/day) and then cases who were SGA (39.54 μg/kg/day), with no statistically significant.

**Fig. 3:**
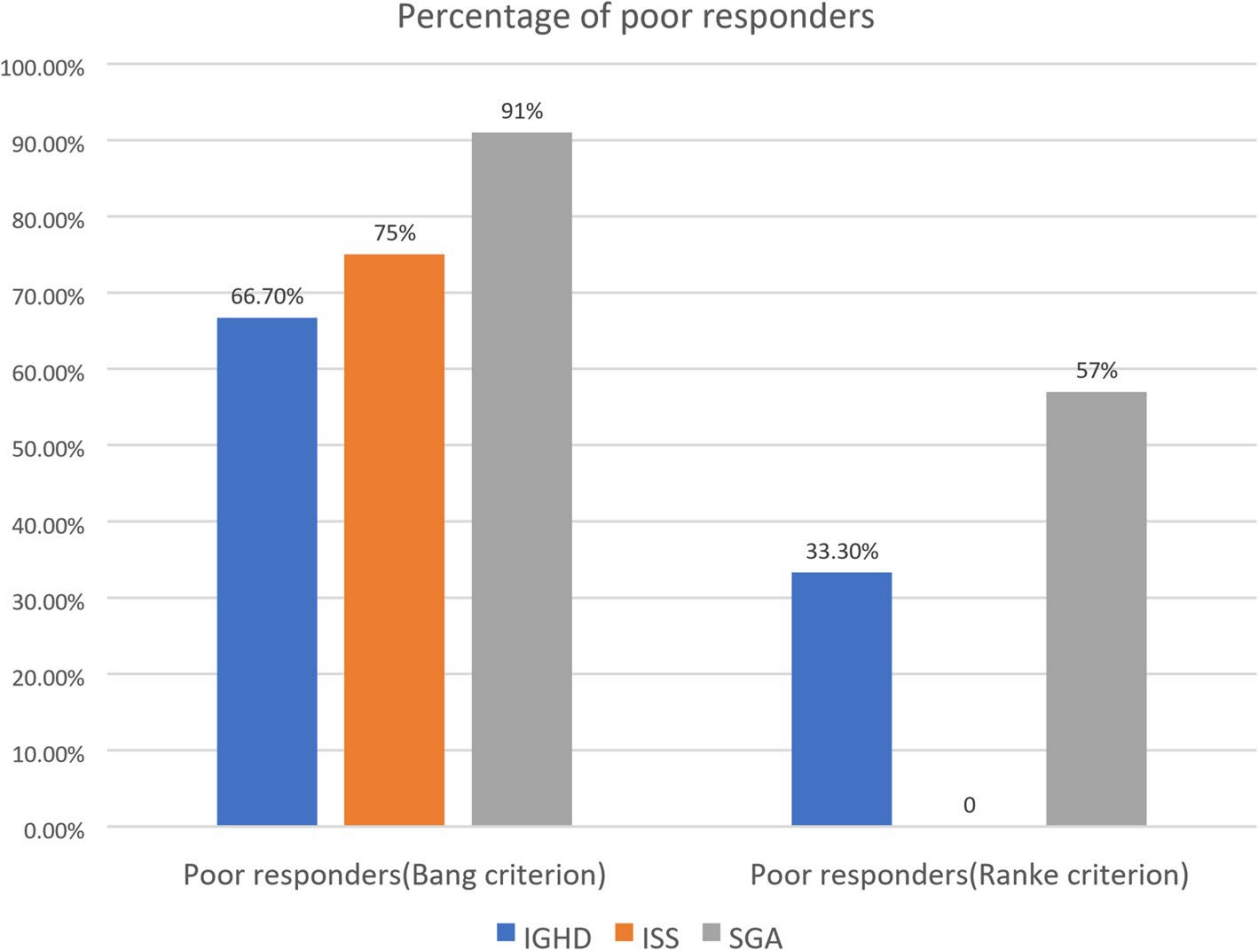
Percentage of poor responders to GH therapy in IGHD, ISS, and SGA cases according to the Bang and Ranke criteria

For evaluation of GH responsiveness, the Bang and Ranke criteria were used (as illustrated in Fig. 3). The majority of cases were poor responders (30 cases, 75% of cases) according to Bang criterion compared to 11 cases (39%) by applying the Ranke criterion.

A good HV is one that falls between − 2 SD and + 2 SD, based on gender and age. Upon correlating height velocity and IGF-1 levels, it was found the majority of cases (53%) exhibited non-matching height velocity and IGF-1 levels (either high height velocity with low IGF-1 or low height velocity with high IGF-1).

## Discussion

This study aimed to describe the pattern of rhGH use and treatment outcomes in children with short stature. In this study, the mean age before starting GH therapy was 12.55 years, the mean initial bone age was 9.27 years (delayed), the mean initial height SD was − 3.42 SD, the mean initial weight SD was − 2.65 SD, and the mean mid-parental height SD was − 0.97 SD. These results were comparable to those of Alzahrani et al. [16] where mean age was 10.77 years ± 2.48 and the mean initial height was 125.35 cm ± 11.72.

The majority of cases (55%) in our study were prepubertal (Tanner stage 1), which is similar to the study of Miller et al. [17].

There was a male predominance in our study as in other studies [18]. This may be related to greater height expectations for boys worldwide. Thus, more parents seek medical advice for their short sons. In spite of this, boys in our study started GH treatment at an older age than the girls (13.26 vs. 12.19 years respectively). These concur with the results of Straetemans et al. [19] where boys also started GH treatment at a significantly older age than girls (7.5 vs. 6.6 years, respectively; *p* = 0.01). This may be explained by the expected earlier growth spurt in girls (girls begin puberty 1 year earlier than boys, so parents are concerned about the height in girls at an earlier age than boys).

In our study, mean height gain (cm/year) was highest in the GHD group followed by ISS then SGA groups. Outcome was less than described by Gahlot et al. [20], where the first-year height velocity in GHD cases was 10.6 ± 3.0 cm/year, despite similar mean chronological age at treatment initiation (12.1 ± 3.1 years). The mean height SD after 1 year treatment in our study was − 3.095 SD in the ISS group, − 3.11 SD in the IGHD group (best response), and − 3.27 SD in the SGA group. These results were similar to the results of Quitmann et al. [1] with mean height SD after 1 year treatment − 2.06 SD in the ISS group, − 1.91 SD in the GHD group (best response), and − 2.05 SD in the SGA group. However, Alzahrani et al. [16] found no significant differences in height gain after treatment between the GHD and ISS groups.

In the present study, only children with IGHD had a median height SDS change ≥ 0.3 SDS following the first year of therapy (0.125 in ISS, 0.38 in IGHD, and 0.06 in SGA). These results were different from results of Al-Abdulrazzaq et al. [21], where all children except ISS group had median height SDS change ≥ 0.3 SDS with the best response appearing in SGA children. Total height SDS gain correlated significantly with pre-pubertal gain in height SDS and younger age at start of treatment in GHD. Early age at start of therapy was also identified as a predictor of adult height in children with ISS.

In order to achieve the optimum height velocity response, the dose of rhGH should be tailored according to GH responsiveness [22]. In the current study, the mean GH dose during the first year of GH treatment was highest among cases with ISS (41.6 μg/kg/day) compared to IGHD (40.79 μg/kg/day) and SGA (39.54 μg/kg/day). The discrepancy between the doses in the current study may be explained by the delayed age of onset of treatment that occurred in a higher percent of IGHD group in comparison to SGA group (86% of IGHD cases started treatment after the age of 10 years, compared to 73% of SGA patients), and ISS usually require highest doses of GH according to literature [23].

Growth response in the first year of rhGH therapy is one of the best indicators of long-term height gain [24]. By applying Bang criterion to our study, the majority of cases (75%) were poor responders [14 cases (66.7%) IGHD, 6 cases (75%) ISS, and 10 cases (91%) SGA]*.* Ranke criterion was applied only on 28 cases; 11 cases only were poor responders (39%) [7 IGHD cases (33.3%) and 4 SGA cases (36.3%)]. These results match those of Straetemans et al. [19] who found that Bang criterion yielded the highest proportion of poor responders was in SGA (37%) and GHD (26%), versus Ranke criterion which yielded only 15% poor responders in SGA and 12% in GHD. Poor response can be explained by the older age at initiation of GH therapy. On the other hand, the results of our study were different from the study of Pozzobon et al. [25] which yielded lower numbers of poor responders (55.3% of patients according to Bang criterion and 23.4% according to Ranke criterion). The lower percentages of poor responders in their study can be explained by the higher initial height SD at start of GH therapy (− 2.42 SD with Bang criterion and − 2.61 SD with Ranke criterion) compared to our study.

The high proportion of poor responders in our study may be explained by older age of starting GH therapy (mean age 13.26 years in boys and 12.19 years in girls) and poor compliance of about one third of cases with the correct dosage and timing of injections, in addition to lack of consistent follow-up that hindered appropriate dose adjustment.

Our study had a number of limitations including heterogeneous nature of the cases, wide age range, and only a small number of the total recruited cases continued follow-up for 1 year. Many cases presented from distant governorates in Egypt, which affected compliance with follow-up and adherence to the proper dosage, especially that recruitment was during the COVID-19 pandemic.

## Conclusion

More males than females seek medical advice for short stature. However, they seek treatment at an older age. IGHD cases had a better height velocity response to GH therapy, followed by ISS then those SGA. Older age for starting GH therapy is associated with poor response to therapy. It is difficult to rely on IGF-1 axis solely in the diagnosis or follow-up of short stature due to many factors including methodology of measurement, reference data, and ranges based on age, gender, and pubertal status.

## Data Availability

The dataset presented in the study is available on request from the corresponding author during submission or after publication. The data are not publicly available due to privacy concerns.

## Abbreviations

DEMPU: Diabetes, endocrine, and metabolism pediatric unit
ELISA: Enzyme-linked immunosorbent assay
GH: Growth hormone
GHD: Growth hormone deficiency
HV: Height velocity
IGF-1: Insulin-growth factor 1
IGHD: Idiopathic growth hormone deficiency
ISS: Idiopathic short stature
ISYS: Information systems
MPH: Mid-parental height
rhGH: Recombinant human growth hormone
SD: Standard deviation
SDS: Standard deviation score
SGA: Small for gestational age
